# The role of regulation in influencing income-generating activities among public sector doctors in Peru

**DOI:** 10.1186/1478-4491-5-5

**Published:** 2007-02-26

**Authors:** Manuel Jumpa, Stephen Jan, Anne Mills

**Affiliations:** 1 Public Health Faculty, Cayetano Heredia University, Lima, Peru; 2 Health Policy Unit, London School of Hygiene and Tropical Medicine, London, UK; 3 Policy and Practice Program, The George Institute for International Health, Sydney, Australia

## Abstract

**Objective:**

To examine in Peru the nature of dual practice (doctors holding two jobs at once – usually public sector doctors with private practices), the factors that influence individuals' decisions to undertake dual practice, the conditions faced when doing so and the potential role of regulatory intervention in this area.

**Methods:**

The study entailed qualitative interviews with a sample of twenty medical practitioners based in metropolitan Lima, representing a cross-section of those primarily employed in either the private or public sectors and engaged in clinical practice or policy making. The interviews focused on:

1. individuals' experience with dual practice;

2. the general underlying pressures that influence the nature and extent of such activities; and

3. attitudes toward, and the influence of, regulation on such activities.

**Results:**

Dual practice is an activity that is widespread and well-accepted, and the prime personal motivation is financial. However, there are also a number of important broad macroeconomic influences on dual practice particularly the oversupply of medical services, the deregulated nature of this market, and the economic crisis throughout the country, which combine to create major hardships for those attempting to make a living through medical practice. There is some support among doctors for tighter regulation.

**Conclusion:**

Research findings suggest appropriate policy responses to dual practice involve tighter controls on the supply of medical practitioners; alleviation of financial pressures brought by macro-economic conditions; and closer regulation of such activities to ensure some degree of collective action over quality and the maintenance of professional reputations. Further research into this issue in rural areas is needed to ascertain the geographical generalizability of these policy responses.

## Background

Dual job holding amongst public sector medical practitioners, where physicians work at the same time in the private sector, is increasingly being recognised internationally as an activity that is widespread and one which regulators have much difficulty in influencing [1-3]. Part of the problem is that in many countries there is a lack of explicit acknowledgement of such activities within their regulatory systems: e.g. in China there are apparently contradictory sets of regulations that exist at national, provincial and hospital levels pertaining to the legality of such activities [4]; in Canada, there is some ambiguity about the legal status of private practice *per se *across provinces [5]; and in Thailand, there are no specific regulations dealing with this issue [6]. Furthermore, in low and middle income countries, there is also often a lack of capacity within government to monitor adequately and implement the regulations that do exist [3,7].

The motivation for individuals to undertake joint public and private activity is usually financial and based on the need to supplement low public sector incomes [2,4,8]. Ethical and legal concerns associated with these practices often arise because the boundaries between individuals' public practice and their outside income generating activities become blurred and consequently such activities are seen to be prone to corruption or unethical behaviour. Two commonly cited problems are the misappropriation of public sector resources and the diversion by a doctor of public patients into his/her private practice [1,3]. Despite this, dual practice has also been shown to have positive aspects. For instance, it provides income opportunities that potentially enable the retention of qualified personnel in public facilities [3,9] and may also increase access to services [10]. Because of these complexities and the problem of low regulatory capacity in many low and middle income settings, crude regulatory responses such as the banning of such activities are often inappropriate and ineffective [3].

A central issue relating to the regulation of dual practice is that it is an activity in which practitioners as individuals tend to resist encroachments on their personal freedom, but collectively the profession has a strong interest in regulation to ensure quality control and prevent oversupply. This phenomenon is consistent with findings from elsewhere [8] and can be characterised as a collective action problem [11,12]. More intense competition in the market for medical services in which dual practitioners are operating tends to exacerbate this discord between collective and individual interest. In exploring options for appropriate and effective regulation of these activities it is therefore important to develop options that are acceptable to providers in order to ensure effective co-operation around compliance, particularly in settings where there is low regulatory capacity within the government.

### Background

The population of Peru as estimated in the 2005 census was 26 million [13]. In 1996 there were 9.8 physicians per 10,000 population although the distribution was uneven across the country. For example, in Lima the rate was 17.3 per 10,000, while in more impoverished areas of the country such as Huancavelica, Apurimac and Cajamarca the rates were 2.8, 2.8 and 3.1 respectively [14].

The health system in Peru broadly comprises three sectors: the Ministry of Health, the Peruvian social security institute (EsSALUD) and the private sector. Private practice in medicine has historically been part of the norm in Peru. Until 1968, Lima's main hospitals were managed by religious orders and were charitable private institutions. In 1968, the national health system was created: hospitals were transformed into being part of the national public health system with doctors within them becoming public servants [15]. However recent official statistics suggest that although 66% of physicians are primarily employed in government institutions, the private sector in physician services has again become more prominent [16].

In 1996, 39% of physicians were primarily employed by the Ministry of Health, 18% by EsSALUD, and 32% in the private sector [17]. It has been estimated that of the 20,000 physicians registered with the Peruvian College of Physicians, around 10,000 held more than one job [18]. This accords with a more recent survey of over 1000 physicians across all regions of the country which found that 57% engaged in some form of secondary income earning activity – 45% of those holding second jobs in the private sector did so as employees of private clinics whiles 36% operated solo practices [19]. Nevertheless the capacity of private hospitals to hire doctors in Peru is constrained by the fact that an overwhelming majority of all health facilities in Peru (81%) are operated by the Ministry of Health [14]. Furthermore, there are prohibitions on Ministry of Health doctors also holding contracts with EsSALUD and vice versa [18].

Although the regulation of medical practice in Peru can be traced back to 1888 [15], much of what presently exists has its origins in the regulatory initiatives that came with the founding of the Ministry of Health (MOH) in 1935 [20]. Recently, the Medical Working Act was passed to regulate medical practice in private and public health centres [21]. As with many countries, however, the regulatory framework of the health system does not have specific provisions in relation to dual practice. The *Ley General de Salud *(health legislation) states that doctors are governed by their professional body in providing medical care [22]. The Peruvian College of Physicians Charter 1968 simply sets out that professionals must observe the ethical rules of medical practice. The most specific reference to dual practice is in the Doctors' Working Law 1990 stating the norms, conditions, hours and salary of Peruvian doctors, but even here there is some degree of vagueness. This legislation seems to provide some scope for dual practice through three articles: the 5^th^, 8^th ^and 14^th^. Article 5 mentions that doctors have freedom to exercise the "medical act" in whatever circumstances. Article 8 rules that "medical acts" are: health care, teaching, management, production and other activities directly related to the medical act. Finally, Article 14 indicates that teaching is permissible simultaneously with health care. The lack of specific provisions dealing with dual practice creates potential for differences in interpretation of the regulations.

Against this background, this paper aims through interviews with doctors to examine the nature of such activities in Peru, determine the manner in which individuals are influenced by prevailing regulatory constraints (or indeed their perceptions of them), and consider options for being able to exercise effective regulatory controls over such activities.

The next section outlines the methods used in the study. This is followed by a presentation of the results. The fourth section discusses the broader implications of the findings (including limitations) and this is followed in the final section by some conclusions.

## Methods

The Peruvian College of Physicians was used as the sampling frame for the study. Although all registered doctors in Peru are listed on this register, due to financial and logistical constraints, only those based in metropolitan Lima were considered. Respondents were sampled purposively to maximise variation by reflecting a cross section of those primarily employed in either the public or private sector; and in clinical practice or policy making. Twenty respondents in total were interviewed before saturation: six of the respondents were from private clinics, five from the Ministry of Health, six from EsSALUD and three were office bearers within the Peruvian College of Physicians. The details of each respondent in terms of these affiliations are highlighted in the reporting of results (below).

Doctors were collected from their place of employment, and interviewed in the Peruvian College of Physicians building. A one-hour session for each, led by a specialist in qualitative studies (although the lead investigator was also present) began with an explanation of the reasons for the study followed by broad questions related to the doctor's professional activities. A step-by-step approach, from the easiest to the hardest questions, was used to progress through the interview stages.

The interviews were built around a series of questions that focused on three key issues:

1. individuals' experience with dual practice;

2. the general underlying pressures that influence the nature and extent of such activities; and

3. attitudes toward, and the influence of, regulation on such activities.

The first and second issues focus on individual behaviour and motivation and the third was about identifies collective interests and potential to achieve co-operative outcomes.

The in-depth interviews were tape-recorded and transcribed in Spanish. The findings were grouped into themes to address the general issues specified above (and summarised in table 1). This process was informed by joint reflections of the interviews from the facilitator and the lead investigator.

**Table 1:** Summary table of results

**Experiences with dual practice**	**Individual motivations**	**Underlying external pressures**	Policy/regulatory levers
Very prevalent	Income	Highly competitive market	**Some in favour of banning**
Popular with younger doctors who tend to be more aggressive	Skills development	Macroeconomic crisis and income pressures	**Tighter workforce planning called for**
Legitimacy based on historical acceptance of DP	Clinical autonomy and access to facilities	Deregulation of medical education	**Adequate public sector income seen as important in reining in uncontrolled dual practice**
Evidence of misuse of public sector resources	Lack of career path and income progression in public sector	Lowering work and pay conditions associated with competition	**Tighter regulation in terms of quality of care**
Favourable outcomes in terms of skills development			
**Emergence of quasi-private clinics within public hospitals**			

## Results

### Individuals' experience with dual practice

Many of the interviewees confirmed the common view that dual practice is widespread amongst public sector doctors:

"The vast majority, almost all doctors working in the public sector, have another job: in their own office or in larger private clinics" (Policy Maker (PM) #2, College of Physicians)

*"It is rare that a doctor has only one job" (PM #2)*.

In terms of whom typically carries out such activities, young male doctors are seen to be the most frequent dual practitioners:

*"They are the more aggressive, because they have less opportunities (in their primary jobs)" (Rheumatologist #16, employed by EsSALUD)*.

*"All doctors have dual jobs, however, junior doctors are increasingly sub-contracted by others" (PM #3, College of Physicians)*.

*"We must divide doctors by generations; the new generation will have options available. However, I would not give up my public job" (Paediatrician #9, Ministry of Health)*.

The main incentive for dual practice is to obtain a better income. Public employment alone fails to offer a comfortable income:

*"The main cause of dual practice is the need for doctors to improve incomes" (PM #2, College of Physicians)*.

Indeed as a result of such pressures, there were frequent reports of doctors holding more than two different jobs to supplement their income.

*"In the past 10 years the classical form of dual practice of a public job in the morning, doctor's office in the afternoon, has been declining; the multi-job practice is replacing it" (PM #1, College of Physicians)*.

Another incentive is the ability to foster and maintain skills in clinical practice. This is very important for junior doctors:

*"Instead of doing nothing at home, or working in non clinical jobs, I prefer clinical practice out of hours even for a minimal wage. I am a doctor and I need to be in touch with patients" (General medicine #,3 private clinic)*.

Other perceived attractions are clinical autonomy and access to better equipment:

"*The private clinics manage their own budget, updated technology and better equipment. This allows the provision of better health care and provides more professional satisfaction" (Othorhinolaryngologist #1, private clinic)*.

Lack of a professional career path/development also has an influence on dual practice. There is the perception that there is no income progression in the public sector and doctors thus need to look for other income sources.

*"There are no professional careers in public institutions. Hospital directors are hand-picked; there is no respect for seniority or postgraduate degrees" (PM #3, College of Physicians)*.

The factors that impact on decisions as to whether to work solely in the public sector or engage in dual practice vary amongst doctors, particularly at differing levels of seniority:

*"For senior doctors it is rather more attractive to have professional improvement and better health equipment in hospitals than more income" (Paediatrician #8, Ministry of Health)*.

In addressing the question of why doctors maintain public sector employment (rather than working exclusively in the private sector) a number of reasons were posited. The income from a job in the public sector was seen to bring security to doctors by offering a monthly salary and social benefits (social security, retirement pension, vacations). This regular income is very important in the insecure employment market in Peru:

*"I wanted to maintain my work at EsSALUD because I needed experience and because they pay better; at least that is what matters, isn't it?" (Gynaecologist #2, EsSALUD)*.

Some expressed a preference for remaining exclusively in the public sector if incomes could be raised to a level which met expectations:

*"I would leave the private job if my public income would pay the equivalent of what I earn now doing extra work teaching, researching and conducting private practice" (Paediatrician #12, EsSALUD)*.

Career improvement and professional challenges were often mentioned in relation to maintaining public employment:

*"It is more intense and challenging working in public hospitals. You improve yourself. Take public paediatric emergency, or surgery in hospitals: you are dealing with many complex cases and scarce resources. You are tested to do your best. It is not routine" (Surgeon #13, EsSALUD)*.

The public job is, however, also considered a useful platform for incursions into the private sector:

*"From my perspective, my public job provides me with social security; from it I can expand my medical activities" (Dermatologist #15, EsSALUD)*.

*"One needs a fixed salary to feel at ease" (Paediatrician #8, Ministry of Health)*.

### Underlying pressures

Many respondents highlighted various work conditions including income and other aspects of the workplace as major concerns for individual doctors.

There has been an increase in competition in the private sector and this has created difficulties for doctors seeking to establish private practice:

*"The problem is there are many doctors and nobody regulates the entrance of more doctors" (Gynaecologist #6, private clinic)*.

As a result, moving into private practice provides no guarantee of financial rewards:

*"Private clinics are nowadays working at 30–40% of their capacity" (Gynaecologist #2, private clinic)*.

*"Establishing a private office is no longer worthwhile. Many of them are now closing down" (Paediatrician #12, EsSALUD)*.

*"Things have changed, and nowadays clinics such as the Hogar de la Madre are declining. Doctors are totally exploited" (Gynaecologist #2, private clinic)*.

The country's macro-economic situation has had a great influence in shaping dual practice:

*"Due to the country's economic crisis, doctors need to find other jobs to survive" (PM #3, College of Physicians)*.

*"Doctors do dual practice because of their domestic requirements (food, children's education, maintain social status), and to maintain their quality of life. Other pressures are the economic crisis... low salaries, fragility of the medical labour system (e.g. doctors on temporary contracts do not have social insurance, do not have professional career incentives); ...oversupply of new doctors leads to more competition and lower fees" (PM #3)*.

Deregulation of medical education also influences dual practice. There are an increasing number of newly graduated professionals:

*"In Lima alone there are seven medicine faculties... and still growing" (General Medicine #3, private clinics)*.

*"One of the problems is the excessive number of specialists. That is because the universities are creating graduates without matching the country needs. Neither the universities nor the MOH actually know how many specialists are needed" (Surgeon #13, EsSALUD)*.

Furthermore, deregulation of medical working conditions has led to a reduction in the number of formally contracted doctors. Increasingly, doctors are employed under temporary contracts with lower wages and no social insurance:

*"Nowadays, being in the medical labour market is precarious; people cannot rely on social security or progressive income increments" (PM #3, College of Physicians)*.

Other factors mentioned are the conditions of work imposed on healthcare suppliers by insurance companies and the role of medical corporations in shaping private practice:

*"The insurance companies have imposed abusive conditions on health suppliers" (Paediatrician #12, EsSALUD)*.

*"Private practice has changed its shape: actually the medical corporations are filling the market. To operate in private practice you now have to fit into the corporation network" (Paediatrician #12)*.

Managerial conditions such as the productivity measures imposed by employers also affect dual practice:

*"It is hard to do dual practice when based at ESSALUD facilities because doctors are required to see a large number of patients per week (averaging around 15 minutes each). However, it is quite easy to do dual practice at the Ministry of Health hospitals since they have not introduced such productivity mechanisms" (Rheumatologist #16, EsSALUD)*.

Young doctors are particularly affected by the private sector's financial problems:

*"Young doctors wait for an opportunity in the private sector, though they are then exploited" (General medicine #3, private clinic)*.

### Attitudes toward, and the influence of, regulation on such activity

Dual practice is seen to have a degree of natural legitimacy that is based on an historical acceptance of such activity:

"There always has been dual practice; it is the main characteristic of medical professional exercise" (PM #1 College of Physicians);

*"The dual practice has always existed" (PM #2 College of Physicians)*.

The use (or, put more bluntly, misappropriation) of public sector resources to subsidise private practice is perceived as a potential problem:

"This is present in public services. Diverse surveys have shown cross subsidies among the Ministry of Health, EsSALUD and the private clinics" (PM #1 College of Physicians);

*"It [misappropriation] is possible; it has not been well studied; However, it is possible" (PM #2 College of Physicians)*.

Some doctors, however, also saw favourable outcomes associated with dual practice:

*"Teaching and clinical practice reinforce each other. Doctors are updated, patients are seen more carefully" (General medicine #11, Ministry of Health)*.

Some doctors interviewed criticised the existence of quasi-private clinics (public facilities that function as private clinics) inside public hospitals. For example, Loayza Hospital in Lima established such a clinic in 1994 allowing patients access to private rooms through payment of consultation fees several times greater than those paid by public patients. This is used as a means of generating private incomes and also generates rental income for the hospital [23]. Though these clinics improve the income of medical staff as well as generating more hospital revenues, they create barriers to patients' access to health care:

*"Patients should have equal access to public facilities; money should not establish differences at government facilities ... these types of private clinics are encouraging self-interest amongst doctors" (General medicine #17, EsSALUD)*.

However, others disagreed:

*"The quality of care is the same for everybody, what changes are the 'hotel' facilities" (PM #2 College of Physicians)*.

*"The quality of care is similar because it is provided by qualified doctors. What makes a difference is the speed of bureaucratic procedures" (PM #3 College of Physicians)*.

There was some support amongst policy makers for banning dual practice, but also some disagreement:

*"Yes, it must be regulated (...) the state must provide for the existence of an exclusive public career (PM #2 College of Physicians)"*.

*"Regulation would be worthwhile if it is linked to developing exclusive dedication to public facilities" (PM #1 College of Physicians)*.

*"I do not think it [banning] is necessary. Doctors have a free right to have a public and a private job. I do not know how we could regulate it. To start, there is no legal framework to do it. Secondly, if we are generating a legal framework, we must address the problem of providing sufficient incomes [in the public sector]" (PM #3, College of Physicians)*.

The state as well as the professional bodies and hospital boards are perceived as necessary for regulation:

*"The state, the Peruvian College of Physicians and the hospital boards, all of them would regulate dual practice" (PM #3, College of Physicians)*.

*"Yes, the Ministry of Health should regulate teaching and clinical practice" (General medicine #3, private clinic)*.

Income, quality of care and workforce planning were three areas for regulation identified:

*"The principal mechanism for regulation is contracting doctors for exclusive dedication to public employment and providing them with an adequate income" (PM #2 College of Physicians)*.

What should be regulated is the quality of care to patients... hospital boards must do it" (Othorhinolaryngologist #1 private clinic);

*"The College must regulate the number of students on each medical faculty" (Gynaecologist #2 private clinic)*.

*"The hospital boards must regulate the number of students for patients, meanwhile the College should regulate the quality of care because it is related to ethics and morals" (Othorhinolaryngologist #1, private clinic)*.

Table 1 provides a summary of the findings.

## Discussion

There is no explicit reference to dual practice in the health sector regulations in Peru and thus its legal status is unclear. Against this background, there is anecdotal evidence highlighting its widespread prevalence. One of the key aims of this study was thus to examine the nature of this activity. The main areas of agreement were that dual practice is seen amongst doctors to be commonplace and furthermore, widely acknowledged. The motivation for such activities is typically financial. In terms of why such doctors continue to maintain their primary roles in the public sector, the main reasons cited were economic security and skills development. Such findings are consistent with those found in other settings [2,4,6,8]. Where there was less agreement was in the nature of the regulatory responses needed around dual practice – some favouring the banning of such activity while others taking a more tolerant approach aimed essentially at minimization of harm.

There are a number of broader features of the Peruvian context that have not been well explored in the studies conducted elsewhere. Firstly, the highly competitive nature of this market in Peru meant that dual medical practice was not seen as necessarily a guarantor of better income. One consequence was the commonly reported need for some to hold more than two jobs. It was found that that younger male doctors tend to be the most aggressive in pursuing dual practice opportunities. Some of the reasons cited for why such pressures are prevalent in Peru were the deregulation of the medical labour market and a lack of workforce planning leading to too many graduates entering the profession. Also, poor macroeconomic conditions have lowered incomes and purchasing power of government employees and diminished their job security. At present, Peru has an unemployment rate of 9.6% but this is masked somewhat by an underemployment rate of 54.9% [24]. Although dual practice has historically been a feature of the Peruvian health care system, such conditions have had the effect of lowering living standards generally and increasing competition in this market and undermining individuals' income earning ability. Another manifestation of these pressures in this market was the 'exploitative' conditions of work many felt were imposed by private employers.

Perhaps not surprisingly, against this background, there was much agreement among medical professionals for greater regulation of such activities, although less agreement as to who should do the regulating. Some believed it should be from government while others advocated some form of self-regulation. One of the key findings of this study is that options for altering the regulations pertaining to dual practice need to be tied in with what is happening in the wider economy and the reforms that affect it. While there was a view expressed that income should be the focus of regulation, there was also acknowledgement of the external pressures on the market for dual practice services and therefore broader regulation of medical education and workforce planning would be considered relevant. This is potentially important in ensuring, at least in the long term, that medical practitioners are able to achieve an adequate standard of living from such activities and preventing the erosion of quality through unfettered competitive pressures in such markets. The key to this is in recognizing that the regulatory issues surrounding dual practice are about medical practice generally rather than dual practice as an isolated activity.

An important aspect of regulation is its potential role in addressing the type of collective action problem created by dual practices in which the individual short term interests of practitioners are not necessarily consistent with the collective interests of the profession. Regulation imposes certain norms of behaviour that may not be achieved more spontaneously through the co-operation of individuals. Such regulations would be required to provide explicit recognition of dual practice activities, specify certain standards of quality that to some extent could be enforced in collaboration with professional bodies such as the College of Physicians, and ensure proper demarcation between activities in the public and private sphere. At the same time, the aim would be to protect professional reputation and preserve some degree of market power. A characteristic of the present situation, as highlighted in the findings, is the deregulated market in medical practice and the difficulties associated with earning a living in such an unrestrained and competitive environment. It has been found elsewhere that in settings where regulatory capacity is low, a collusion of interests can seriously undermine the implementation of a piece of regulation [25]. Therefore ensuring that regulation is designed so that there is at least the perception of a 'win-win' situation amongst key stakeholders can be important in ensuring proper implementation – particularly where a lack of regulatory capacity is likely to be a major constraint on government action. In other words, successful regulation in this area requires the doctors themselves to want to be regulated. The findings of this study suggest that such conditions for collective action exist in this setting.

One limitation of the study is that the findings were drawn from metropolitan based doctors where it is likely that competition for the provision of medical services is most intense. It is perhaps less likely to be so amongst rural doctors. This may have a bearing on the generalizability of these findings and therefore, until further studies are conducted in rural areas of Peru, any policy response may need to be specific to metropolitan areas rather than national. The difficulty with qualitative approaches to studying these issues is that generalizability cannot be specifically tested. In this study we have tried to ensure broad coverage of issues by examining the differing perspectives generated through our purposive sampling strategy.

Elsewhere it has been found that opportunities for additional income earning opportunities are better for medical specialists rather than generalists [6]. This study did not specifically seek to address whether this was the case in Peru.

Another limitation of the approach taken is that by examining only the views of doctors (albeit from different sectors), it provides a somewhat partial perspective of the phenomenon of dual practice and one possibly clouded by self-interest. In focusing on this perspective, the study is not able to address the normative questions relating to the rights and wrongs of dual practice. Rather, it is a study more of regulatory scope and seeks to build an understanding of the appropriate levers which need to be pulled in order to effect change in the behaviour of individual actors. In this sense, it is not a study simply of dual practice, but of the regulatory context and the regulatory mechanisms to which various actors respond.

## Conclusion

This study highlights the importance of understanding the broader macroeconomic and regulatory context in which an activity such as dual practice occurs, in order to establish an appropriate set of policy options. Regulation of these activities is problematic without recognition of the forces that push individuals into them and shape the conditions in which they are carried out. The evidence suggests that there is recognition amongst doctors that dual practice is a consequence of system issues and that regulatory responses thus need to be as far-reaching. In Peru, some of the options that could be considered are tighter controls on the supply of medical practitioners, measures to alleviate financial pressures brought by macro-economic conditions, and closer regulation of doctors engaging in dual practice to enforce some measure of collective action. Importantly, in framing an appropriate and effective policy response, the study has highlighted the role of regulation in reconciling the collective interests within the medical profession with broader policy objectives such as maintaining staff in the public sector and quality control.
